# Raw Single-Wall Carbon Nanotubes Induce Oxidative Stress and Activate MAPKs, AP-1, NF-κB, and Akt in Normal and Malignant Human Mesothelial Cells

**DOI:** 10.1289/ehp.10924

**Published:** 2008-05-16

**Authors:** Maricica Pacurari, Xuejun J. Yin, Jinshun Zhao, Ming Ding, Steve S. Leonard, Diane Schwegler-Berry, Barbara S. Ducatman, Deborah Sbarra, Mark D. Hoover, Vincent Castranova, Val Vallyathan

**Affiliations:** 1 Health Effects Laboratory Division, National Institute for Occupational Safety and Health, Morgantown, West Virginia, USA; 2 Department of Pathology, School of Medicine, West Virginia University, Morgantown, West Virginia, USA; 3 Division of Respiratory Disease Studies, National Institute for Occupational Safety and Health, Morgantown, West Virginia, USA

**Keywords:** asbestos, cancer, carbon nanotubes, cell injury, DNA damage, mesothelioma, nanoparticles

## Abstract

**Background:**

Single-wall carbon nanotubes (SWCNTs), with their unique physicochemical and mechanical properties, have many potential new applications in medicine and industry. There has been great concern subsequent to preliminary investigations of the toxicity, biopersistence, pathogenicity, and ability of SWCNTs to translocate to subpleural areas. These results compel studies of potential interactions of SWCNTs with mesothelial cells.

**Objective:**

Exposure to asbestos is the primary cause of malignant mesothelioma in 80–90% of individuals who develop the disease. Because the mesothelial cells are the primary target cells of asbestos-induced molecular changes mediated through an oxidant-linked mechanism, we used normal mesothelial and malignant mesothelial cells to investigate alterations in molecular signaling in response to a commercially manufactured SWCNT.

**Methods:**

In the present study, we exposed mesothelial cells to SWCNTs and investigated reactive oxygen species (ROS) generation, cell viability, DNA damage, histone H2AX phosphorylation, activation of poly(ADP-ribose) polymerase 1 (PARP-1), stimulation of extracellular signal-regulated kinase (ERKs), Jun *N*-terminal kinases (JNKs), protein p38, and activation of activator protein-1 (AP-1), nuclear factor κB (NF-κB), and protein serine-threonine kinase (Akt).

**Results:**

Exposure to SWCNTs induced ROS generation, increased cell death, enhanced DNA damage and H2AX phosphorylation, and activated PARP, AP-1, NF-κB, p38, and Akt in a dose-dependent manner. These events recapitulate some of the key molecular events involved in mesothelioma development associated with asbestos exposure.

**Conclusions:**

The cellular and molecular findings reported here do suggest that SWCNTs can cause potentially adverse cellular responses in mesothelial cells through activation of molecular signaling associated with oxidative stress, which is of sufficient significance to warrant *in vivo* animal exposure studies.

By 2015, the worldwide market for products with nanotechnology components will reach an estimated $1 trillion (Roco 2005). The unique behavior and properties of nanoscale materials have revolutionized technology, producing an estimated 1,300 materials either in use or being tested for potential commercial applications. Enhanced physical and chemical properties associated with the nanosize of these materials have been exploited to produce a wide variety of new products. In addition, nanoparticles are being explored for several treatment modalities, including early detection of tumors and other clinical applications (Gwinn and Vallyathan 2006). With these applications come unprecedented avenues of human exposure to nanomaterials. Engineered single-wall carbon nanotubes (SWCNTs) are a class of nanoparticles being actively evaluated for myriad industrial and biomedical applications (Dresselhaus et al. 2004). Exponential growth in the use of SWCNTs potentially can cause exposure to a large number of workers (Maynard 2007).

SWCNTs have been reported to have many adverse cellular and animal toxicity reactions, which may be predictive of detrimental human health effects upon exposures (Lam et al. 2004; Shvedova et al. 2005). The likely widespread industrial application of SWCNTs in several consumer products and medical applications may pose an emerging human health concern (Donaldson et al. 2006; Maynard 2007). It has been suggested that inhaled SWCNTs and other nanoparticles are likely to evade phagocytosis, penetrate lung tissue, and translocate to other organs to cause systemic cell toxicity and injury (Gwinn and Vallyathan 2006; Oberdörster et al. 2005). Therefore, toxicity studies of nanoparticles should not be limited to a single lung cell or only to the lung, but should involve other systemic targets.

Preliminary cellular and animal exposure investigations on toxicity and pathogenicity of SWCNTs have demonstrated biological interactions, including toxicity, inflammatory reactions, oxidative stress, and fibroproliferative response (Lam et al. 2004; Mercer et al. 2008; Shvedova et al. 2005). SWCNTs are biopersistent and have the ability to distribute to subpleural areas after pharyngeal aspiration (Mercer et al. 2008). These earlier investigations compelled the present studies of potential interactions of SWCNTs with mesothelial cells.

Epidemiologic, animal, and cellular studies indicate that exposure to crocidolite asbestos (crocidolite) can cause pulmonary fibrosis, lung cancer, and malignant mesothelioma (Manning et al. 2002). Data indicate that mesothelioma in 80–90% of individuals is associated with crocidolite as the primary cause. Because mesothelial cells are the primary target cells of asbestos-induced molecular changes mediated through an oxidant-linked mechanism, we used well-characterized SWCNTs with a known concentration of metal catalyst contamination to investigate the alterations in molecular signaling in response to SWCNT exposure in normal mesothelial (NM) and malignant mesothelial (MM) cells. We used this form of SWCNTs because SWCNTs with different redox-sensitive iron contents have displayed diverse redox potentials, with iron-rich SWCNTs causing a significant loss of glutathione and increased lipid peroxidation in alveolar macrophages (Kagan et al. 2006).

In this study we examined the toxicity and alterations in molecular signaling pathways in mesothelial cells exposed to raw SWCNTs with significant metal contamination. We compared some results with known effects of crocidolite in cultured mesothelial cells.

## Materials and Methods

### SWCNT particles

We obtained commercially manufactured raw SWCNTs through collaboration with the National Institute of Standards and Technology (Gaithersburg, MD); a more detailed description is presented in the Supplemental Material available online at http://www.ehponline.org/members/2008/10924/suppl.pdf. Reported impurities include nickel and yttrium, which are encapsulated in carbon shells (see Supplemental Material, Table 1). Detailed high-resolution transmission electron microscopy revealed that the diameters ranged from 0.8 to 2.0 nm (see Supplemental Material, Figure 1C).

### Mesothelial cell culture

Exposure to crocidolite is well documented to cause mesothelioma in humans and animals, and cellular studies using mesothelial cells are reported to mimic important biologic responses involved in mesothelioma development. Therefore, in the present study we used normal human NM and malignant MM mesothelial cells that we maintained as described in the Supplemental Material (available online at http://www.ehponline.org/members/2008/10924/suppl.pdf).

### Electron spin resonance (ESR) assay

We determined the production of reactive oxygen species (ROS) caused by exposing NM and MM cells to SWCNTs as described in the Supplemental Material (available online at http://www.ehponline.org/members/2008/10924/suppl.pdf).

### Intracellular detection of O_2_^•−^ and H_2_O_2_ in intact cells by confocal microscopy

We investigated intracellular production of ROS generation in mesothelial cells exposed to SWCNTs, crocidolite, or vehicle [RPMI-1640 medium containing 0.1% fetal bovine serum (FBS)]. We used the dyes dihydro-ethidium (DHE) and dichlorodihydro-fluorescein diacetate (H_2_DCFDA) for the intracellular localization of O_2_^•−^and H_2_O_2_ and the intracellular detection of ROS as described in the Supplemental Material (available online at http://www.ehponline.org/members/2008/10924/suppl.pdf).

### Cell viability assay

We seeded the NM and MM cells (5 × 104) overnight and treated them with 12.5, 25, or 125 μg/cm^2^ SWCNTs or with vehicle alone for 24 hr. We evaluated cell viability using 3-[4,5-dimethylthiazolyl-2]-2,5-diphenyltetrazolium bromide (MTT) assay kit according to the manufacturer’s instructions (Roche Molecular Biochemicals, Indianapolis, IN), and as described in the Supplemental Material (available online at http://www.ehponline.org/members/2008/10924/suppl.pdf).

### DNA damage by comet assay

We seeded the NM and MM cells (10^5^) overnight and treated them with vehicle or 25 or 50 μg/cm^2^ SWCNTs for 24 hr. We assessed DNA damage using a commercially available comet assay according to the manufacturer’s instructions (Trevigen, Gaitherburg, MD) and as described in the Supplemental Material (available online at http://www.ehponline.org/members/2008/10924/suppl.pdf).

### Histone H2AX phosphorylation of DNA double-strand breaks

We exposed NM and MM cells cultured in black-wall/clear-bottom microplates to 25 or 50 μg/cm^2^ SWCNTs or crocidolite for 24 hr. We detected H2AX phosphorylation according to the manufacturer’s protocol (Millipore, Billerica, MA) and as described in the Supplemental Material (available online at http://www.ehponline.org/members/2008/10924/suppl.pdf).

### Western blot analysis of cleaved poly(ADP-ribose) polymerase (PARP)

We subcultured NM and MM cells and maintained them overnight in 10% FBS growth medium. We then replaced the standard growth medium with 0.1% FBS-containing medium; exposed the cells to 50 μg/cm^2^ SWCNTs or crocidolite for 0, 6, or 18 hr; and analyzed PARP activation as described in the Supplemental Material (available online at http://www.ehponline.org/members/2008/10924/suppl.pdf).

### Protein kinase phosphorylation assay

We treated NM and MM cells (10^6^) seeded overnight with 25 μg/cm^2^ SWCNTs or vehicle for varying times up to 120 min and assayed phosphorylation as described in the Supplemental Material (available online at http://www.ehponline.org/members/2008/10924/suppl.pdf).

### Activation of activator protein-1 (AP-1) and nuclear factor κB (NF-kB)

We seeded the NM and MM cells (10^6^) in six-well plates overnight and treated them with 25 μg/cm^2^ SWCNTs or vehicle for 1, 2, or 4 hr. We prepared nuclear extractions using a nuclear extraction kit and determined activation of AP-1 and NF-κB using an enzyme-linked immunosorbent assay (ELISA) kit (Panomics Inc., Redwood City, CA) according to the manufacturer’s instructions.

### Western blot analysis of protein serine-threonine kinase (Akt)

We cultured NM and MM cells (2 × 105) in six-well plates with 10% FBS medium. We then washed the cells and exposed them to 0, 25, 75, or 125 μg/cm^2^ SWCNTs in 0.1% FBS for 30 or 60 min. We analyzed Akt phosphorylation as described in the Supplemental Material (available online at http://www.ehponline.org/members/2008/10924/suppl.pdf).

### Statistics

Data presented are mean ± SE of values compared and analyzed using one-way analysis of variance. We considered *p* ≤ 0.05 statistically significant.

## Results

Although SWCNTs are not water soluble, in the present investigation we used a vehicle containing 1% FBS and ultrasonication to suspend the SWCNTs for cell exposure studies. Light microscopy, scanning electron microscopy, and transmission electron microscopy studies of the suspended samples showed that this technique produced homogeneous dispersion of SWCNTs with small agglomerates of nanoropes and mats of SWCNTs [see Supplemental Material, Figure 1A–C (available online at http://www.ehponline.org/members/2008/10924/suppl.pdf)].

### Trace metals in SWCNTs

Among the 31 metals analyzed in three different SWCNT samples, we identified metals suspected of having toxic biological effects [see Supplemental Material, Table 1 (available online at http://www.ehponline.org/members/2008/10924/suppl.pdf)]. Two redox-sensitive trace metals (iron, 0.07%; nickel, 20.6%)—in appreciable concentrations—and yttrium (6.2%) were present in raw SWCNTs.

### ROS generation by SWCNT-exposed mesothelial cells

Generation of ROS monitored by ESR in SWCNT-exposed NM and MM cells revealed that the NM cells generated more ROS than did MM cells [see Supplemental Material, Figure 2A, center panel (available online at http://www.ehponline.org/members/2008/10924/suppl.pdf)]. The reaction mixture with the cells in the absence of SWCNTs did not produce any detectable ESR signals, whereas addition of SWCNTs produced a distinct ESR signal spectrum with cells [see Supplemental Material, Figure 2A, center panel). The hyper-fine splitting of the spin adduct produced by SWCNTs were characteristic evidence of hydroxyl radical (•OH) generation.

Exposure of NM and MM cells to 500 μg/mL SWCNTs significantly increased •OH radical generation [see Supplemental Material, Figure 2B (http://www.ehponline.org/members/2008/10924/suppl.pdf)], which was higher in NM cells than in MM cells (see Supplemental Material, Figure 2B). When catalase, a decomposing enzyme of H_2_O_2_, was present, the SWCNT-induced ESR signal 5,5-dimethyl-1-pyrroline-1-oxide (DMPO)-•OH was inhibited by 39% in NM cells (*p* < 0.05) and by 43% in MM cells [see Supplemental Material, Figure 2A, right panel; Figure 2B). Deferoxamine, a metal iron chelator, produced a similar inhibition pattern in both cell types. The SWCNT-induced ESR signal intensity decreased 38% in NM cells and 44% in MM cells (see Supplemental Material, Figure 2B). This indicates that some chelatable metals are partially involved in the generation of SWCNT-induced •OH radicals. However, because the chelation did not completely nullify the generation of ROS, the cell stimulation by SWCNTs could be considered a potential source of ROS generation. We present data on semiquantitative measurement of differential DMPO-•OH signal intensities and inhibition induced by catalase and deferoxamine in the Supplemental Material (Figure 2B).

### Intracellular detection of ROS generation in intact cells

**Figure 1:**
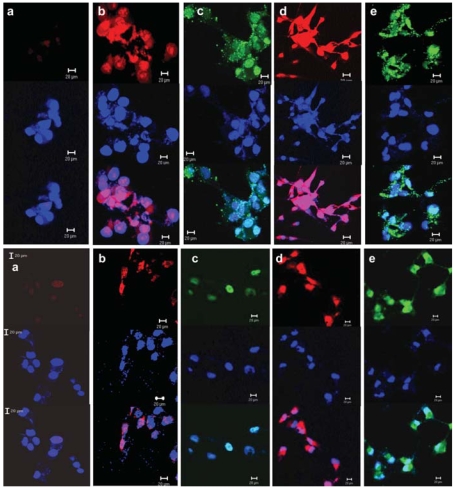
Confocal micrographs of O_2_^•−^ and H_2_O_2_ generation in intact NM cells (top) and MM cells (bottom) treated with SWCNTs and crocidolite. a, control; b and c, SWCNT; d and e, crocidolite; b and d, DHE staining; c and e, H_2_DCFDA staining. Red, localization of O_2_^•−^; green, localization of H_2_O_2;_ blue, diamidinophenylindole. Bars = 20 μm.

To further confirm the ability of NM and MM cells to generate ROS after exposure to SWCNTs and crocidolite, we analyzed the cells treated with particles by intracellular staining for O_2_^•−^ and H_2_O_2_. DHE (a specific fluorescent dye for O_2_^•−^), and H_2_DCFDA (a fluorescent dye specific for H_2_O_2_) were used to monitor ROS generation. In the presence of 150 μg/mL SWCNTs for 90 min, the fluorescence for O_2_^•−^ and H_2_O_2_ were increased in both NM and MM cells. ROS generation was much greater in NM cells than in MM cells, confirming the ESR studies (Figure1). Fluorescence for O_2_^•−^ and H_2_O_2_ was significantly greater with crocidolite than with SWCNTs in both cell types (Figure 1). Catalase or superoxide dismutase (SOD) pre-treatment abolished most of the particle-induced generation of ROS (data not shown).

### Effects of SWCNTs on cell viability

We evaluated the effects of SWCNTs and crocidolite on cell viability of NM and MM cells using MTT, lactate dehydrogenase (LDH), and trypan blue assays. However, LDH enzyme molecules were adsorbed by the SWCNTs, and only crocidolite caused a strong cytotoxicity in increasing doses (data not shown). Therefore, only MTT and trypan blue viability assay results are presented for comparison of cytotoxicity [see Supplemental Material, Figure 3A,B (available online at http://www.ehponline.org/members/2008/10924/suppl.pdf)]. Cell viability studies using the MTT assay indicated that SWCNTs in increasing mass concentrations of 12.5, 25, and 125 μg/cm^2^ (incubated for 24 hr at 37°C) caused a dose-dependent decline in cell viability in both NM and MM cells. The SWCNT-dependent decrease in cell viability was significant compared with control samples in both cell types (see Supplemental Material, Figure 3A). The trypan blue exclusion assay showed that exposure of both cell types to mass doses similar to those used for the MTT assay also caused decreasing cell viability with increasing doses. However, cell viability by trypan blue assay was significantly decreased only with the two higher doses of 50 and 125 μg/cm^2^ SWCNTs (see Supplemental Material, Figure 3B). Exposure of NM and MM cells to 12.5, 25, and 50 μg/cm^2^ crocidolite for time points similar to those for SWCNTs resulted in a dose-dependent decrease in cell viability, and the decrease was significantly greater compared with SWCNT samples over the tested doses. A concentration of 50 μg/cm^2^ crocidolite decreased cell viability by 75–77%, whereas SWCNTs at the same concentration and exposure time decreased cell viability by only 30% and 27%, for NM and MM cells, respectively (see Supplemental Material, Figure 3C). We also obtained similar results for crocidolite in the MTT cell viability assay (data not shown).

### DNA damage induced by SWCNTs

**Figure 2:**
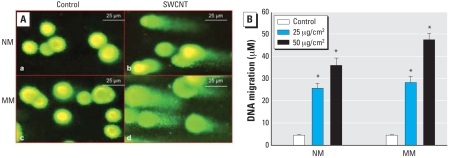
Effect of SWCNTs (25 or 50 μg/cm^2^) on DNA migration in NM and MM cells using the comet assay. (*A*) Micrographs of NM (a,b) and MM (c,d) cells treated with vehicle (control; a,c) or 50 μg/cm^2^ SWCNTs (b,d) for 24 hr: (*B*) Semiquantitative analysis of concentration-dependent effects of SWCNTs on DNA migration in NM and MM cells using the comet assay. Data are presented as the mean ± SE of three experiments. Bars = 25 μm *Significant increase from control (*p* < 0.05).

Because SWCNT exposure caused generation of ROS, we investigated whether raw SWCNT-induced oxidative stress resulted in DNA damage. DNA damage, investigated using a comet assay in NM and MM cells exposed to 25 or 50 μg/cm^2^ SWCNTs or vehicle for 24 hr, showed that the SWCNTs induced DNA damage in both cell types (Figure 2A). Figure 2B shows semiquantitative measurements of SWCNT-induced dose-dependent DNA tail migration, demonstrating significant DNA damage at both doses in NM and MM cells (Figure 2B). Exposure of NM cells to 25 or 50 μg/cm^2^ SWCNTs for 24 hr resulted in a 5.2- and 6.6-fold increase in DNA tail length migration, respectively. In contrast, exposure of MM cells to the same mass concentrations of crocidolite for 24 hr caused an increased DNA tail migration of 7.9- and 11.1-fold, respectively. Coincubation of MM cells with SWCNTs (25 μg/cm^2^) and catalase (100 U/mL), SOD (100 U/mL), or deferoxamine (1 mM; an iron chelator) for 24 hr resulted in a 35%, 30%, and 32% decrease in DNA damage, respectively.

### H2AX phosphorylation by SWCNTs and crocidolite

**Figure 3:**
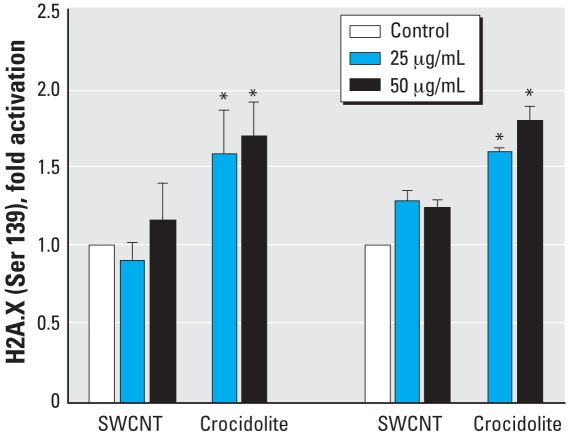
Effect of 24-hr exposure to SWCNTs or crocidolite (25 or 50 μg/mL) on the activation of γ-H2AX (Ser139) in NM and MM cells. Absorbance values were normalized to control values; data are presented as mean ± SE of three experiments *Significantly different from control (*p* ≤0.05).

Exposure of NM and MM cells to 25 or 50 μg/cm^2^ SWCNTs resulted in a nominal increase in phosphorylation of H2AX on Ser139, which was moderately higher in MM cells. The same concentrations of crocidolite induced a significantly greater phosphorylation in both cell types (Figure 3).

### Effects of SWCNTs and crocidolite on PARP

**Figure 4:**
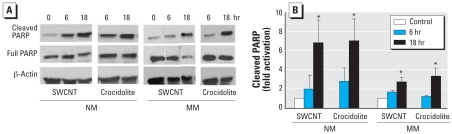
Effects of SWCNTs and crocidolite on the activation of PARP in NM and MM cells. (*A*) Cells were exposed to 50 μg/cm^2^ SWCNTs or crocidolite for 6 or 18 hr and then examined by Western blot analysis for cleaved PARP (each blot is from one representative experiment per treatment). Detection of full PARP and β-actin of the same membrane ensured equal sample loading. (*B*) Results of densitometric analysis of PARP activation to total PARP; the fold activation is relative to normalized values of unstimulated control specimens. Data shown are mean ± SE of three experiments *Significantly different from control (*p* ≤ 0.05).

Apoptosis is often associated with PARP cleavage, leading to the activation of caspase; therefore, we investigated the effects of exposure to SWCNTs or crocidolite in NM and MM cells. PARP, a chromatin-bound enzyme activated by DNA strand breaks, may alter the chromosomal proteins to facilitate DNA repair. Our studies with SWCNTs and crocidolite show time-dependent activation of cleaved PARP in NM cells. Crocidolite and SWCNTs induced significantly greater activation of PARP in NM cells compared with MM cells (Figure 4). MM cells showed moderate activation of cleaved PARP after 18 hr exposure to SWCNTs or to crocidolite. SWCNTs caused only a 2-fold activation after 6 hr compared with 2.8-fold activation by crocidolite (Figure 4). These results indicate that enhancement of DNA repair is significantly impaired in MM cells.

### Effect of SWCNT exposure on AP-1 and NF-κB activation

**Figure 5:**
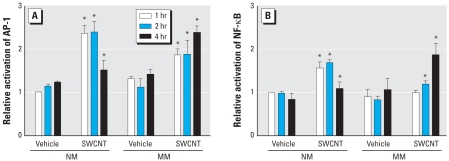
ELISA assay results showing the effect of 1-, 2-, or 4-hr treatment with 25 μg/cm^2^ SWCNTs on the activation of AP-1 (*A*) and NF-κB (*B*) in NM and MM cells. Data shown are the mean ± SE of three experiments *Significant increase from controls (*p* < 0.05).

We examined effects of SWCNT-induced ROS on the activation of redox-sensitive signaling pathways, especially the activation of two important transcription factors, AP-1 and NF-κB. AP-1 was activated in NM cells incubated with 25 μg/cm^2^ SWCNTs in the first 1–2 hr and then declined after 4 hr (Figure 5A). On the other hand, in MM cells, the same mass concentration of SWCNTs induced a smaller early response, with an activation similar to that seen in NM cells only after 4 hr (Figure 5A).

Exposure of NM and MM cells to 25 μg/cm^2^ SWCNTs resulted in a similar response in the activation of NF-κB (Figure 5B). In NM cells, the NF-κB activation was maximal at 1–2 hr and then declined by 4 hr. In MM cells, a time-dependent peak response of NF-κB activation was achieved only after 4 hr. This delayed activation of NF-κB in MM cells was 2-fold greater than the basal level at 4 hr.

### Effect of SWCNT exposure on mitogen-activated protein kinase (MAPK) phosphorylation

**Figure 6:**
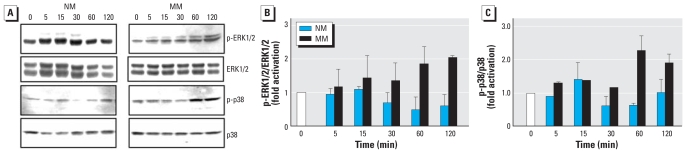
Effect of SWCNTs on the activation of MAPKs in NM and MM cells. Abbreviations: p-p38, phosphorylated p38. (*A*) Western blot analysis of NM and MM cells treated with vehicle or 25 μg/cm^2^ SWCNTs for 5–120 min showing phosphorylated and nonphosphorylated ERK1/2 and p38. (*B*) Densitometric analysis of Western blots of p-ERK1/2 signal normalized to total ERK1/2. (*C*) Densitometric analysis of Western blots of p-p38 signal normalized to total p38. Fold activations are relative to normalized values of unstimulated cells; data shown are mean ± SE of three experiments.

Because MAPKs are the upstream kinases responsible for c-Jun phosphorylation and AP-1 and NF-κB activation, we investigated which classes of MAPKs are activated by the SWCNTs. We examined the effects of SWCNTs on phosphorylation of extracellular signal-regulated kinase 1/2 (ERK1/2), Jun *N*-terminal kinases (JNKs), and protein p38 kinase in NM and MM cells. Treatment of MM cells with SWCNTs led to increased phosphorylation of ERKs and p38 (Figure 6A) but not JNKs (data not shown). Alterations in the phosphorylation of ERKs and p38 in NM cells were very minimal and occurred only at the 15-min time point, where both p38 and phosphorylated ERK1/2 (p-ERK1/2) showed significant phosphorylation in MM cells compared with NM cells (Figure 6B,C). The studies using Western blot and densitometry clearly demonstrate the significant difference in response of MM cells at 60 and 120 min (Figure 6B,C). These results indicate that activation of AP-1 and NF-κB by SWCNTs may be mediated through the induction of ERKs or p38 signaling in NM and MM cells.

### Effect of SWCNTs on Akt

**Figure 7:**
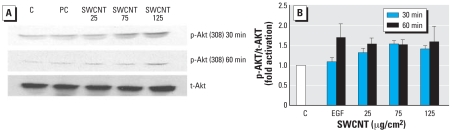
Activation of p-Akt (Thr308) induced by SWCNTs. Abbreviations: C, control; PC, positive control (EGF, 40 ng/mL). (*A*) Western blot analysis of Akt in MM cells treated with 25, 75, or 125 μg/cm^2^ SWCNTs for 30 or 60 min in medium containing 0.1% FBS; detection of total Akt of the same membrane was used to ensure equal sample loading per lane. (*B*) Densitometric analysis of Western blots showing p-Akt signal normalized to total Akt (t-Akt). The fold activation is relative to normalized values of unstimulated control specimens; data shown are mean ± SE of four experiments.

Involvement of Akt, a signal transduction protein regulated by downstream signaling by phosphoinositide 3-kinase (PI-3K), is reported to play a major role in lung tumor and mesothelioma genesis. Because of this important role of Akt in tumorogenesis, we examined the association between SWCNT exposure and activation of Akt in NM and MM cells. Western blot analysis of Akt (Thr308) after 30 and 60 min of exposure of NM and MM cells to SWCNTs induced activation of phosphorylated Akt (p-Akt) only in MM cells to the level slightly lower than the positive control, epidermal growth factor (EGF) (40 ng/mL) (Figure 7A). This activation was dose and time dependent at 30 min of SWCNT exposure and then remained at the same level after 60 min (Figure 7A). Densitometric analysis indicates a 1.6-fold increase by 125 μg/cm^2^ SWCNTs compared with 1.7-fold increase by EGF at 60 min in MM cells (Figure 7B). In NM cells, p-Akt was not expressed after exposure to the same or higher concentrations of SWCNT.

## Discussion

Several studies have shown that occupational and environmental exposures to particulate matter with mean diameters of < 10 μm or nanoparticles with a size dimension < 100 nm are associated with respiratory diseases, including cancer (Knaapen et al. 2004). The present study focused on raw SWCNTs, which represent one of the most widely investigated nanoparticles with enormous potential for industrial, technologic, and medical applications. SWCNTs have been shown to translocate to subpleural areas in the lung, and therefore may have the potential to cross the cell membrane to the mesothelial layer (Mercer et al. 2008). Although SWCNTs have been the subject of extensive research over the last few years, their potential interactions with human mesothelial cells have not been reported. Because they behave like asbestos, with biopersistence and ability to generate ROS, the potential human health impacts and risks compel us to understand the toxic and molecular interactions reported here. These studies are further justified by a recent report on the induction of mesothelioma in p53^+/−^mice by multiwall carbon nanotubes (Takagi et al. 2008). The data presented in the present study provide basic information regarding the potential health hazard of SWCNTs and support the bioactivity of SWCNTs on mesothelial cells *in vitro*, although with lower levels of activity compared with crocidolite.

Asbestos fibers have high aspect ratios, are biopersistent, and contain high concentrations of iron with the potential to generate ROS. These characteristic fiber features, combined with the ability to translocate to mesothelium, are well documented as major contributing factors triggering the development of mesothelioma by asbestos, leading to the activation of cell signaling pathways, early response genes, and carcinogenesis (Manning et al. 2002). Because raw SWCNTs used in this study also have a high aspect ratio, have high metal contamination (nickel, yttrium, iron), are biopersistent, and are reported to translocate to subpleural areas in the lungs, the present investigation is warranted. SWCNT exposures to animals indicate that SWCNTs are not well recognized by macrophages and that the pulmonary inflammatory response to SWCNTs is not persistent, yet progressive interstitial fibrotic response has been noted (Kagan et al. 2006; Lam et al. 2004; Shvedova et al. 2005). Mercer et al. (2008) reported deposition of labeled SWCNTs to the distal alveolar interstitium including subpleural areas and mesothelium. Therefore, transport of SWCNTs from the distal airspaces to the pleura and/or extrapulmonary locations, including mesothelium, is a possibility. The present study indicates that SWCNTs are toxic to NM and MM cells. However, the degree of toxicity of SWCNTs was one-third that of crocidolite in NM and MM cells [see Supplemental Material, Figure 3B,C (available online at http://www.ehponline.org/members/2008/10924/suppl.pdf)].

Oxidative molecular mechanisms triggered by the persistent ability of asbestos fibers to cause injury to the mesothelial cells have been reported to be features involved in asbestos-fiber–induced mesothelioma development (Heintz et al. 1993). Cellular reactions observed in animal models, mesothelial cell lines, and patients with crocidolite-induced mesothelioma are reported to be similar (Altomare et al. 2005a; Kane 2006; Ramos-Nino et al. 2006; Vaslet et al. 2002). Therefore, recapitulation of biochemical and molecular events observed in human mesothelial cells exposed to raw SWCNTs reported in the present study may provide a functional basis to explore the potential of SWCNTs to induce mesothelioma in animal models.

The ability of engineered nanomaterials to interact with biological tissues and generate ROS has been proposed as possible mechanisms involved in the toxicity (Nel et al. 2006). ROS are well known to play both a deleterious and a beneficial role in biological interactions. Oxidative damage due to ROS results in damage to DNA, proteins, and lipids and in the activation of cell signaling pathways that are associated with loss of cell growth regulation, leading to carcinogenesis (Valko et al. 2006). In the present study, raw SWCNTs upon interaction with NM and MM cells induced the formation of ROS, as demonstrated by ESR as well as *in situ* localization. The level of raw SWCNT-dependent •OH radicals generated was approximately 1.6-fold higher in NM cells than in MM cells. *In situ* localization of ROS confirmed the ESR data and provided parallel results indicating greater generation of ROS in NM cells by the interaction of both SWCNTs and crocidolite. The increased generation of ROS caused by exposure to particles has been shown for many different forms of fine, ultrafine, and nanoscale particles, including SWCNTs, to be associated with minimal metal contamination (Sharma et al. 2007; Shvedova et al. 2003).

Nanoparticles, because of their physical and chemical properties, are unique compared with known fine-sized parent compounds that behave differently in toxicity and DNA damage (Knaappen et al. 2004). Also, the mechanism of particle-induced DNA damage could be direct or indirect and is not fully understood. Genotoxic effects may be produced either by direct interaction of particles with genetic material or by secondary damage from particle-induced ROS. Biopersistence of particles and the potential to translocate through the lung to the mesothelium is a major contributing factor involved in sustained ROS generation and DNA damage of mesothelial cells. In the present study, addition of ROS scavengers resulted in a moderate reduction of the extent of DNA damage and not in a complete abrogation of the damage. These results suggest that genotoxic effects of SWCNTs occur in part through direct damage to DNA and not solely through oxidative stress. In addition, asbestos-like fibrous characteristics of SWCNTs are likely to contribute to the mechanisms involved in a sustained level of ROS generation inducing DNA damage. In support of this potential of SWCNTs to generate ROS, Kisin et al. (2007) demonstrated that exposure of lung fibroblast V79 to acid-purified SWCNTs also resulted in DNA damage. The present results and the existing literature therefore suggest that the genotoxic effects of SWCNTs result from a combination of direct effects of crocidolite-like behavior and the potential of metal catalysts associated with SWCNTs to induce oxidative stress on a sustained basis. However, the exact genotoxic mechanism of SWCNTs *in vitro* remains to be elucidated. ROS in the absence of anti-oxidant protection can directly interact or modify cellular proteins, lipids, and DNA, which in turn may alter cellular functions and predispose the cells to impaired apoptosis and abnormal cell growth. Continued oxidative stress induced by these mechanisms may disrupt DNA repair, cause mutations, and change growth patterns and gene expression. These events are well documented in animals exposed by intraperitoneal injections of crocidolite and have been linked to the development of malignant mesothelioma (Goodglick et al. 1997; Vaslet et al. 2002). Although ROS have been linked to the development of mesothelioma, the exact mechanisms by which mesothelial cells are transformed to malignant cells by asbestos are not fully understood. Animal and cellular studies show that asbestos fibers induce mesothelioma by direct interaction of the fibers with the mesothelial cells and the generation of ROS, which in turn promotes signaling and activation of cascades of events that may finally induce cancer.

H2AX phosphorylation is a very rapid and sensitive response to DNA damage and occurs within a short time after exposure to ionizing radiation and environmental stress (Redon et al. 2002). As a result of the DNA double-strand breaks, the histone H2AX protein can be distinguished from other histones by a unique carboxy-terminal sequence that is rapidly phosphorylated at the fourth residue (Ser139) in response to DNA damage (Rogakou et al. 1998). H2AX phosphorylation occurs rapidly irrespective of the type of DNA damage, resulting in the phosphorylation of thousands of H2AX molecules (Pilch et al. 2003). The detection of H2AX phosphorylation and the induction of double-strand breaks induced by SWCNTs and crocidolite complement other molecular evidence supporting potential carcinogenic activity. However, H2AX phosphorylation by SWCNTs was less than that observed with crocidolite exposure.

In this study, we observed a dramatic time-dependent increase in cleaved PARP after exposure to SWCNTs and crocidolite at 6 and 18 hr in NM cells. This high activation of PARP can lead to depletion of ATP and nicotinamide adenine dinucleotide levels and cell death in NM cells. Similar concentrations of SWCNTs or crocidolite in MM cells caused relatively lesser activation of cleaved PARP at 6 and 18 hr. The enzymatic activation of PARP is induced when DNA damage occurs and PARP protein is cleaved during apoptosis to signal the repair pathways that contribute to posttranslational modification of histones and nuclear proteins (Huber et al. 2004). The higher levels of ROS seen by ESR and *in situ* localization, as well as the increased activation of PARP in NM cells by SWCNTs, support the potential to induce transformation of NM cells.

A growing body of evidence suggests that EGF, platelet-derived growth factor, and the insulin signal transduction pathway mediated by PI-3Ks play important roles in the activation of Akt. PI-3K/Akt has been shown to be associated with carcinogenesis, and this signal pathway is important in cell survival and proliferation (Nicholson and Anderson 2002). The PI-3K phosphorylated membrane phospholipid products induce the activation of Akt. Akt-1 is an important modulator of insulin signaling and cell proliferation, and Akt-2 plays a major role in cell survival (Heron-Milhavet et al. 2006). In malignant mesothelioma cells and many other types of human cancers, Akt is constitutively activated and is reported to play important roles in the development and aggressiveness of mesothelioma (Altomare et al. 2005b; Nicholson and Anderson 2002). Akt is also a redox-sensitive target for oxidant and growth factor stimulation, such as hepatocyte growth factor and its receptor tyrosine kinase, c-Met, which are highly expressed in mesotheliomas through the activation of PI-3K/Akt pathway (Ramos-Nino et al. 2008). Akt activation may also be involved with the functional changes in tumor suppressor genes involved in the pathogenesis of mesothelioma. Elevated levels of Akt trigger antiapoptotic events and activation of NF-κB, angiogenesis, telomerase activity, and tumor metastasis. Current evidence also suggests that growth-promoting genes, such as c-*fos* and c-*jun*, are activated by these signaling mechanisms that are involved in the development of mesothelioma (Heintz et al. 1993).

Induction of oxidative stress by raw SWCNTs containing redox active iron has been reported in human keratinocyte (HaCaT) cells (Shvedova et al. 2003). This oxidative assault to cells could lead to cytotoxic responses or even cell death via an apoptotic pathway or by necrosis (Higuchi 2004). ROS are involved in activation of AP-1 and NF-κB, and both transcription factors play an important role in carcinogenesis (Ding et al. 2001). AP-1 is one of the transcription factors involved in the oxidative stress response after changes in cellular oxidative status. In this respect, AP-1 has been identified as a target of the MAPK family, including ERKs, JNKs, and p38 kinase (Karin et al. 1997). In addition, the activation of NF-κB has been shown to be regulated by some upstream MAPKs that regulate JNK activation in the cells (Ding et al. 2001).

In summary, the cellular studies reported here clearly demonstrate that NM cells are more susceptible to raw SWCNT-induced injury. Exposure of mesothelial cells to raw SWCNTs resulted in the generation of •OH, leading to several molecular alterations, activation of important signaling pathways, and transcription factors. Such responses are similar to reported asbestos-induced changes common in animal and human mesothelioma development. In this study, we found that *in vitro* exposure of NM and MM cells to SWCNTs altered molecular pathways associated with carcinogenesis. However, uncertainty lingers as to whether SWCNT exposure is a risk for mesothelioma development in humans. To address this question, *in vivo* animal studies are warranted. In human mesotheliomas, deletions of the *Cdkn2a/Arf* and *Cdkn2b* gene loci associated with hypermethylation are reported to be common at the *NF2* gene locus (Kane 2006). Because heterozygous *Nf2*+/− mice exposed to crocidolite develop malignant mesothelioma at a faster rate than wild-type littermates, this mouse model could be used as an ideal and relatively rapid animal model to study the potential of SWCNTs to cause mesothelioma.
